# The Evolving Applications of Creatine Supplementation: Could Creatine Improve Vascular Health?

**DOI:** 10.3390/nu12092834

**Published:** 2020-09-16

**Authors:** Holly Clarke, Do-Houn Kim, Cesar A. Meza, Michael J. Ormsbee, Robert C. Hickner

**Affiliations:** 1 Department of Nutrition, Food and Exercise Sciences, Florida State University, Tallahassee, FL 32306, USA; hec17e@my.fsu.edu (H.C.); dkim5@fsu.edu (D.-H.K.); cm18dq@my.fsu.edu (C.A.M.); mormsbee@fsu.edu (M.J.O.); 2 Department of Biokenetics, Exercise and Leisure Sciences, School of Health Sciences, University of KwaZulu-Natal, Westville 4041, South Africa; 3 Institute of Sports Sciences and Medicine, Florida State University, 1104 Spirit Way, Tallahassee, FL 32306, USA

**Keywords:** creatine, cardiovascular disease, vascular health, oxidative stress, inflammation

## Abstract

Creatine is a naturally occurring compound, functioning in conjunction with creatine kinase to play a quintessential role in both cellular energy provision and intracellular energy shuttling. An extensive body of literature solidifies the plethora of ergogenic benefits gained following dietary creatine supplementation; however, recent findings have further indicated a potential therapeutic role for creatine in several pathologies such as myopathies, neurodegenerative disorders, metabolic disturbances, chronic kidney disease and inflammatory diseases. Furthermore, creatine has been found to exhibit non-energy-related properties, such as serving as a potential antioxidant and anti-inflammatory. Despite the therapeutic success of creatine supplementation in varying clinical populations, there is scarce information regarding the potential application of creatine for combatting the current leading cause of mortality, cardiovascular disease (CVD). Taking into consideration the broad ergogenic and non-energy-related actions of creatine, we hypothesize that creatine supplementation may be a potential therapeutic strategy for improving vascular health in at-risk populations such as older adults or those with CVD. With an extensive literature search, we have found only four clinical studies that have investigated the direct effect of creatine on vascular health and function. In this review, we aim to give a short background on the pleiotropic applications of creatine, and to then summarize the current literature surrounding creatine and vascular health. Furthermore, we discuss the varying mechanisms by which creatine could benefit vascular health and function, such as the impact of creatine supplementation upon inflammation and oxidative stress.

## 1. Introduction

Creatine is an organic compound that is both synthesized endogenously and found exogenously in various food sources such as meats and fish. Since creatine’s isolation and extraction from animal skeletal muscle by French chemist Michel Eugène Chevreul in 1832, the function of creatine has been extensively researched. Furthermore, studies such as those by Chanutin [1], Walker [2], and Harris et al. in 1992 [3] have all shown that supplemental creatine can augment natural human intramuscular creatine stores. These studies, among others, pioneered the current understanding of creatine and the use of creatine supplementation to promote energy provision and to benefit skeletal muscle performance and health. Considering the undeniably important role that creatine and phosphocreatine (PCr) play in rapid energy provision, it is of no surprise that the primary focus of creatine research has centered around the ergogenic effects of creatine supplementation to improve exercise performance. The accumulation of creatine-focused research has contributed to a vast body of knowledge and has led to several authors declaring creatine as being one of the most effective and underrated nutritional supplements [4,5,6]. Furthermore, with clear scientific support and expanding mainstream popularity, creatine remains one of the most dominant sports supplements on the market, accumulating more than $400 million in annual sales [7,8].

In addition to the well-known ergogenic value of creatine [5,9], there has been an emerging interest in the clinical application of creatine. Creatine has been cited as a potential adjuvant therapy for the treatment of a variety of diseases such as myopathies, dystrophies, inflammatory diseases, neurodegenerative disorders, metabolic disturbances, and joint syndromes [4]. With advancing understanding, it is clear that the function of creatine goes far beyond that of a primary role in metabolism and energetics. In fact, recent evidence indicates that creatine supplementation results in a multitude of non-energy-related beneficial effects on a wide range of cellular targets. Among these promising effects includes the antioxidant potential of creatine, scavenging and neutralizing the reactive oxygen species (ROS) that underly many pathologies [10,11].

Despite the scientific literature supporting the use of creatine for performance enhancement and for the potential treatment of pathologies, the possible application of creatine supplementation for the improvement of vascular health has not yet been fully examined. With this being said, some studies have elucidated that creatine supplementation may be able to attenuate factors such as homocysteine [12,13,14], inflammation [15,16,17], and damaging ROS [10,11,18]; all of which, if left uncontrolled or circulating in augmented amounts, have been associated with heightened cardiovascular disease (CVD) risk and compromised vascular health [19,20,21]. Therefore, despite the scarcity of literature focusing on creatine within the vasculature, there may be mechanisms modulated by creatine that can be linked to vascular health. In addition to this gap in the creatine literature, the exact mechanisms by which creatine exerts non-energy-related benefits are still relatively unknown; therefore, there is a need for further research into the more novel applications and functions of creatine.

The aim of this short review is to briefly outline the cellular role of creatine and to touch upon the potential therapeutic application of dietary creatine supplementation in clinical populations. We will then summarize the current literature available on creatine supplementation and its effects upon the vasculature specifically. Furthermore, we will highlight the possible mechanisms by which creatine could therapeutically promote vascular health, exploring creatine’s impact upon homocysteine, inflammation, and oxidative stress.

## 2. Brief Overview of Creatine Metabolism and the Cellular Actions of Creatine

**Figure 1:**
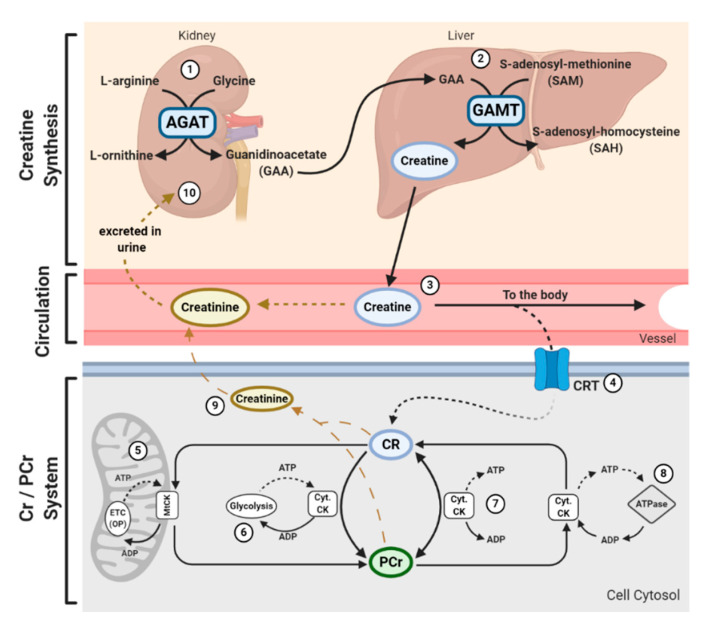
Physiological Journey of Creatine: Synthesis of creatine (Cr) happens at a rate of ~1 g/day [7] via an interorgan process. (1) Within the kidneys, l-arginine: glycine amidinotransferase (AGAT) transfers an amidino group from L-arginine to glycine, resulting in the formation of L-ornithine and guanidinoacetate (GAA). (2) GAA is then transferred and processed in the liver. Guanidinoacetate N-methyltransferase (GAMT) transfers a methyl group from the methyl donor S-adenosylmethionine (SAM) to GAA, resulting in the formation of Cr and S-adenosylhomocysteine (SAH). SAH can thereon be hydrolyzed into homocysteine by S-adenosylhomocysteine hydrolase (not shown). (3) Cr is released from the liver into circulation, where Cr is transported to varying tissues such as the skeletal muscle, brain, kidney, and heart. (4) Cellular uptake of Cr is mediated by a creatine transporter (CRT), or *SLC6A8*. Cr carries both positive and negative charges, and is transported via secondary-active transport, driven by a sodium/chloride-ATPase generated gradient. Once in the cell, Cr has a multitude of fates. (5) Cellular Cr can be transformed into phosphocreatine (PCr) by mitochondrial creatine kinase (mtCK) which is coupled to oxidative phosphorylation (OP) via the electron transport chain (ETC). (6) Cr can be converted into PCr by cytosolic creatine kinase (Cyt. CK) coupled to glycolysis. (7) The cellular Cr/PCr pool is utilized to maintain adenosine triphosphate (ATP)/ adenosine diphosphate (ADP) ratios through ATP resynthesis or “buffering.” (8) Cyt. CKs located throughout the cytosol can utilize the high-energy PCr stores to shuttle and utilize energy at sites of ATP demand, or ATP-dependent processes, via ATPase enzymes. Such processes include ATP-gated ion channels, ATP-regulated receptors, ATP-regulated ion pumps; contractile processes, cell motility, cell signaling, or organelle transport. (9) Both Cr and PCr are naturally metabolized into creatinine via a non-enzymatic, spontaneous reaction. Creatinine diffuses freely into the circulation to be transported to the kidneys. (10) Creatinine is fully excreted in the urine.

In addition to being consumed in an omnivorous diet, creatine is also synthesized endogenously. Endogenous synthesis of creatine is an interorgan process and requires the investment of three major amino acids: glycine, arginine, and methionine; together with two primary enzymes: l-arginine: glycine amidinotransferase (AGAT) and guanidinoacetate N-methyltransferase (GAMT). The first step of creatine biosynthesis occurs in the kidneys, when AGAT catalyzes the transfer of an amidino residue from arginine to glycine, resulting in the formation of l-ornithine and guanidinoacetate (GAA). GAA then exits the kidneys and is transported to the liver where GAMT functions to transfer a methyl group from S-adenosylmethionine (SAM) to GAA, resulting in the final production of creatine. Cellular uptake of creatine is mediated by a specific creatine transporter (CRT), also known as SLC6A8. This transporter is sodium- and chloride-dependent, requiring at least two sodium ions and one chloride ion for the transport of one creatine molecule [22]. Given its vital role in metabolism and energy provision, the largest stores of creatine are found in skeletal muscle (~95%); however, other notable stores include the brain, kidneys, and liver [5]. Intracellularly, creatine can exist in a free form or in a phosphorylated form, PCr. Both creatine and PCr are metabolized and lost naturally throughout the day via a non-enzymatic, spontaneous reaction into creatinine, which is then excreted at a rate of ~2 g/day by the kidneys in the urine [23,24,25] (Figure 1).

Creatine and PCr, together with creatine kinase (CK) isoenzymes, function as quintessential high-energy compounds crucial for metabolism. In the case of low adenosine triphosphate (ATP) levels or high ATP demand, CK will catalyze the transfer of the *N*-phosphoryl group from PCr to adenosine diphosphate (ADP) to resynthesize ATP. This process quickly replenishes the ATP pool, maintaining ATP:ADP ratios and cellular homeostasis. Conversely, when ATP production from either glycolytic or oxidative pathways are greater than ATP utilization, CK can function in reverse to capture and store this cellular energy by replenishing PCr stores. There is a long-held belief that the primary function of the creatine-phosphocreatine system (Cr-PCr system) is to serve as a temporal high-energy phosphate buffer [23,26] (Figure 1). The presence of specific CKs throughout the cell are integral to the function of the Cr-PCr system. CKs exist in a variety of isoforms, which, in addition to the subcellular distribution and compartmentalization of such CKs, led to the proposal that the Cr-PCr system plays a far more complex role in energy metabolism than once believed. Cytosolic CKs (Cyt.CKs) exist as dimers, composed of either muscle (*M*) type or brain (*B*) type; therefore, three cytosolic isoenzymes exist: muscle-muscle creatine kinase (MM-CK); brain-brain creatine kinase (BB-CK); and, muscle-brain creatine kinase (MB-CK) [27]. Specific mitochondrial CKs (MtCKs) also exist, such as sarcomeric MtCK (sMtCK) found in striated muscle and ubiquitous MtCK (uMtCK) found in other tissues such as the brain [28]. MtCKs are found between the inner and outer mitochondrial membranes, and when in the presence of creatine, ensure the bulk of ATP from oxidative phosphorylation is converted into PCr [23,28]. Cyt.CKs, on the other hand, are found within the cytoplasm and at sites of high energy consumption or demand (e.g., cellular ATPases, myofibrils, sarcoplasmic reticulum, plasma membrane) [23]. With a sophisticated variety of CKs and their subcellular localization, the Cr-PCr system is capable of functioning as an energy shuttle of high energy phosphates, shuttling energy between sites of mitochondrial ATP production and sites of ATP utilization [26] (Figure 1).

The function of the Cr-PCr system as a temporal high-energy phosphate buffer and a spatial high-energy shuttle are not mutually exclusive and coexist in varying degrees. The masterful interplay between both shuttle and buffering abilities enables the Cr-PCr system to intricately monitor and stabilize ATP:ADP ratios within the cell, minimize adenine nucleotide loss, maintain cellular pH via hydrogen ion buffering, and to reduce free inorganic phosphates [29,30,31]. Furthermore, it has been speculated that it is the interaction between MtCKs and Cyt.CKs that ensures the maintenance of ATP:ADP ratios within the mitochondrial matrix, thereby stimulating healthy respiratory chain function [32]. This therefore leads to a reduction in electron leakage and reduced production of harmful mitochondrial-specific ROS.

From the above, it is clear that the Cr-PCr system plays a vital role in cellular function. Those readers interested in expanding their knowledge on the function, compartmentalization, and pharmacokinetics of the Cr-PCr system are directed to read reviews by Wallimann et al. [33] and Perksy and Brazeau [34].

## 3. Pleiotropic Application of Creatine

Creatine supplementation has been widely utilized by healthy individuals and athletes as an ergogenic aid to improve intermittent high-intensity exercise capacity due to the Cr-PCr system sustaining rapid ATP resynthesis. The total creatine pool (creatine + PCr) of a 70 kg individual is approximately 120 mmol/kg of dry muscle mass, or around 60–80% saturation [5]. Pivotal research conducted by Dr. Roger Harris and colleagues demonstrated that oral creatine supplementation is capable of increasing muscle creatine and PCr stores by around 20% [3]. Although consumed naturally in the diet (~1–2 g/day) and synthesized daily, with intramuscular creatine metabolism and excretion of around 2 g/day [23], additional dietary supplementation with creatine analogs, such as creatine monohydrate, remains the most efficient way of increasing creatine stores. A common supplementation protocol usually involves a loading phase of 4 × 5 g of creatine for five to seven days, followed by a maintenance phase of 3 to 5 g/day [3,5]. A low dose protocol of consuming 3 g/day for approximately 28 days, however, can still result in increased intramuscular creatine stores [3]. Considering the extensive body of literature on the efficacy of creatine supplementation, there is evidence that creatine supplementation can enhance many exercise-related variables such as exercise capacity [35,36,37,38], recovery [5,39,40], resistance to fatigue [41,42,43], and lean body mass [5,44], in both young and older individuals. For in-depth reviews highlighting the ergogenic value of creatine supplementation, readers are directed to those by Kreider et al. [5] and Butts et al. [8].

With significant success as an ergogenic aid, the potential application of creatine supplementation in clinical populations has gained attention. Creatine supplementation has been shown to impart a variety of benefits upon skeletal muscle, such as the enhancement of force output during skeletal muscle contraction [45], the augmentation of lean body mass [46], fatigue resistance [41,42], and the improvement of intracellular calcium handling [47]. Furthermore, it has been proposed that creatine supplementation may impart further favorable effects on skeletal muscle physiology and metabolism, such as enhancing growth and hypertrophy through direct modulation of components of the mammalian target of rapamycin (mTOR), secretion of myokines such as myostatin and insulin-like growth factor-1, and increasing the expression of myogenic regulatory factors which can stimulate satellite cell mitotic activity [48,49,50]. Considering the beneficial impact of creatine upon muscle, several investigators have studied the effects of creatine on myopathies. Low creatine and PCr stores have been observed in those suffering from muscular disorders, contributing to poor cellular bioenergetics and muscle integrity [51]. These findings led to the proposed hypothesis that creatine supplementation may serve as a therapeutic intervention for myopathies. Tarnopolsky and Martin supported this hypothesis, reporting substantial increases in muscle strength, exercise capacity, and body mass in patients with mitochondrial cytopathies, neuropathic disorders, dystrophies/congenital myopathies, and inflammatory myopathies [52]. Further trials by Tarnopolsky et al. [53], Walter et al. [54], and Louis et al. [55] provide additional evidence that supports the use of creatine for the therapeutic management of various myopathies; however, the complexity and difference between myopathies has limited the ability to make an overarching conclusion. In addition, the potential therapeutic application of creatine for neurological diseases has similarly been hypothesized. The brain, despite having a relatively small mass, represents one of the largest sources of energy consumption, accounting for approximately 20% of resting metabolism [56,57]. While all energy systems play a vital role in ATP provision, the presence of brain-specific CKs suggests a vital role of the Cr-PCr system within the brain [58,59]. Furthermore, considering genetic creatine deficiency syndromes are often characterized by cognitive impairment, developmental delay, autistic behavior, and seizures, it is clear that creatine contributes to healthy brain function [60]. Researchers have since tested this hypothesis and have shown that creatine supplementation can aid in the improvement of cognitive processes such as memory and attention in both young [61] and older individuals [62,63]. It has also been shown that creatine exhibits potential anti-depressant properties [64]. Animal and in vitro models have further been used to assess the efficacy of creatine supplementation for certain neurodegenerative disorders such as Parkinson’s, Huntington’s, and Alzheimer’s disease, some of which report promising results in regard to offering neuroprotection against oxidative stress and neurotoxicity [65,66,67]. Results of clinical trials, however, remain mixed, with some trials reporting potential benefit following creatine supplementation that warrants further investigation [68,69,70,71], and others reporting minimal or no benefits [72,73].

The physiological benefits of creatine do not stop at the muscular and neurological systems. Creatine supplementation has also been found to help ameliorate hyperglycemia [74] and improve glycemic control in those suffering from type 2 diabetes [75], improve function in those suffering from fibromyalgia [76], protect the integumentary system from age-related deterioration and damage [77], increase bone mineral density and tensile strength in elderly individuals [78], decrease triglyceride accumulation and increase liver health in models of non-alcoholic fatty liver disease [79], and protect both mitochondrial and nuclear deoxyribonucleic acid (DNA) from markers of oxidative and inflammatory damage [11,18,80].

Despite the above described potential of creatine for the management of various metabolic, muscular, and neurological diseases, there is surprisingly very little information on the use of creatine supplementation to reduce the current leading cause of mortality in the United States (US): CVD. Major examples of CVDs can include coronary heart disease, heart failure, stroke, atherosclerosis, hypertension, and peripheral artery disease. It has been estimated that approximately 610,000 deaths are caused by CVDs in the US every year [81], with more than 43.7 million adults aged >60 years suffering from one or more CVDs in 2016 alone [82]. Deteriorations in vascular integrity such as arterial thickening, stiffening, endothelial dysfunction, and inflammation are associated with most CVDs, and are all related to, or augmented by, the accumulation of ROS [83,84,85,86]. Considering creatine’s proposed antioxidant properties and promising application within varying clinical populations, the sparse amount of research on the effect of creatine supplementation on vascular function and health is surprising and highlights a major gap in the literature. The following sections of this review outline the current literature available on creatine supplementation and vascular health and function, in addition to possible mechanisms by which creatine may improve vascular health.

## 4. Current Clinical Trials of Creatine Supplementation and Vascular Health

The maintenance of healthy vasculature is vital for longevity; which is in part why the development of vascular pathologies remains the leading cause of mortality in the US. In this respect, maintenance of normal endothelial function has a significant influence on overall vascular health. Endothelial cells (ECs) line the innermost layer of vessels and contribute to the intricate control of the vascular system. The vascular endothelium adapts to humoral [87], mechanical [88], and neural stimuli [89]. In addition, the endothelium plays a role in fluid filtration and control of vasomotor tone through release of vasoactive factors such as nitric oxide (NO), regulation of blood flow and blood pressure, hemostasis, hormone trafficking, angiogenesis, immune response, and inflammation [90,91]. Like many biological targets, ECs can be damaged by varying factors such as ROS [92] and chronic inflammation [93], both of which can be augmented by, or a result of, hyperglycemia [94], dyslipidemia [95], obesity [96], smoking [97], alcohol abuse [98], and aging [99]. This damage can result in deleterious alterations to endothelial physiology, consequentially leading to endothelial dysfunction (ED). ED can be characterized by a reduction in the bioavailability of the potent vasoactive compound NO [100,101], often resulting in impaired endothelium-dependent vasodilation, which is associated with CVD [85]. In addition, ED can lead to ECs expressing more procoagulation factors, promoting a prothrombotic, pro-inflammatory state [90], all of which underly a multitude of CVDs [102]. While pharmacological approaches may be effective in attenuating or managing vascular deteriorations seen with CVD, side effects, financial costs, and medical adherence often hinder these benefits, with many older adults reporting poor adherence due to fear of side effects and prescription costs [103]. This is in part why the consumption of dietary nutritional supplements, or the use of nutraceuticals, has increasingly grown in popularity and is utilized by many older individuals for the maintenance and promotion of health and wellbeing [104].

Despite the promising potential of creatine as an adjuvant therapy or nutraceutical, there have been few studies investigating the role of creatine in vascular health. Among the few to investigate whether creatine supplementation could influence vascular function were Arciero et al. [105]. Arciero et al. investigated whether creatine, either alone or in combination with resistance training (RT), could enhance lower limb (calf) and forearm blood flow. Thirty healthy male volunteers were assigned to receive creatine alone (4 × 5 g/day for 5 days, followed by 2 × 5 g/day for 23 days); creatine + RT; or placebo (maltodextrin 4 × 5 g/day for 5 days, followed by 2 × 5 g/day for 23 days) + RT. It was reported that following creatine supplementation, limb blood flow in both the calf and forearm increased significantly; however, these changes were only seen in the creatine + RT group and not in the creatine alone or placebo groups. The authors stated that these findings indicated a synergistic interplay, or “additive” effect, between creatine and RT. These findings are similar to that of other studies that suggest the benefits of supplements are often augmented when used in combination with physical activity [106,107], but provide little evidence to suggest creatine could independently impact vascular function in younger, healthy adults.

Sanchez-Gonzalez et al. [108] sought to determine whether creatine alone could help improve hemodynamic and vascular responses to a short bout of isokinetic exercise in young healthy males. Subjects ingested either a placebo (maltodextrin) or creatine (2 × 5 g/day) for three weeks. Brachial systolic blood pressure (SBP), heart rate (HR), brachial-ankle pulse wave velocity (baPWV), and leg pulse wave velocity (PWV) were measured at rest before and after the intervention, in addition to 5 and 15 min after exercise. The authors reported that creatine attenuated the increase in SBP seen 5 min after exercise, and HR at both 5 and 15 min after exercise. The ingestion of creatine also led to suppressed increases in baPWV and faster return to resting hemodynamics. It was concluded that creatine supplementation may have improved the hemodynamic and vascular responses to isokinetic exercise by reducing left ventricle afterload and reducing muscle ammonia and lactic acid production, which would have otherwise stimulated sympathetic-mediated increases in heart rate and blood pressures [109]. Considering that PWV is an indicator of arterial stiffness [110] and that heart rate recovery time is a powerful indicator of mortality [111], these findings are in contrast to those of Arciero et al. and suggest a potential benefit of creatine supplementation for vascular function and health.

Two further studies, by Moraes et al. [112] and Van Bavel et al. [12], assessed the impact of creatine supplementation more specifically on the microvasculature. Moraes et al. investigated the effect of creatine (20 g/day for one week) on systemic microcirculation, microvascular reactivity, and skin capillary density in healthy males [112]. Following supplementation, it was reported that creatine significantly increased skin functional capillary density and recruitment during post-occlusive reactive hyperemia. It was further reported that cutaneous microvascular vasodilation induced by hyperemia increased following creatine supplementation. Despite the lack of a control group for true comparison, the improvement shown here in vascular reactivity holds great promise. As previously mentioned, the ability of ECs to release vasoactive compounds and to control vasomotor tone is paramount for the management of blood flow and blood pressures. The inability of ECs to release vasoactive compounds, such as NO, or to stimulate endothelium-derived hyperpolarization factors (EDHFs) to induce vasodilation, is a primary underlining characteristic of many vascular pathologies [113,114]. Moraes et al. demonstrated that following creatine supplementation, vascular reactivity significantly improved. Although no direct mechanism of action for creatine was reported, it was speculated by the authors that creatine may have contributed to an increased epoxyeicosatrienoic acid (EET) bioavailability and thereon improved EDHF stimulation and microvascular dilation [115]. However, this claim was not supported by any literature and again no mechanism was proposed. Alternatively, it was proposed that the increase in intracellular creatine levels caused by supplementation may have been sufficient to activate ATP-dependent potassium channels, thereby hyperpolarizing vascular smooth muscle cells and enhancing hyperemia-mediated vasodilatation.

Van Bavel et al. similarly assessed the effects of creatine on the systemic microcirculation, although they studied subjects who were habitually using a strict vegan diet [12]. Considering dietary creatine is found primarily in meat and fish sources, it has been shown that vegans present with lower creatine stores than those ingesting an omnivorous diet; hence the speculation that vegans, or vegetarians, may benefit from creatine supplementation to a greater extent [116]. Forty-nine vegan subjects were separated into either creatine (5 g/day for three weeks) or placebo (5 g/day maltodextrin for three weeks) groups. Laser speckle imaging with acetylcholine (ACh) skin iontophoresis was used to measure cutaneous microvascular reactivity, and intravital video-microscopy was used to determine skin capillary density and reactivity at rest and following post-occlusive reactive hyperemia. Basal capillary density of the creatine group was found to be significantly increased in comparison to the placebo group following supplementation. Further supporting the findings of Moraes et al., the authors also reported a significant increase in capillary recruitment during post-occlusive reactive hyperemia for those in the creatine group, but not the placebo group. Despite these promising results, the authors did not assess any specific mechanism of action in which creatine may have produced these benefits. The authors did, however, report that creatine supplementation led to a decrease in plasma homocysteine in hyperhomocysteinemic subjects, which may have yielded the improvements seen. It was further proposed that a reduction in vascular oxidative stress may have also resulted in vascular benefits; however, biomarkers of vascular oxidative stress were not directly assessed.

To our knowledge, the studies mentioned above remain the only studies to date that have utilized specific vascular methodologies such as plethysmography, PWV, and microscopy to investigate the impact of creatine on micro- or macro-vascular function. These previous findings reported by Sanchez-Gonzalez et al. [108], Moraes et al. [112], and Van Bavel et al. [12] suggest a possible application of creatine to improve vascular function; however, the lack of a broader body of knowledge warrants the need for further investigation of creatine use to improve vascular function. One commonality shared between these studies that should be considered is the use of healthy, young individuals as study subjects. Considering that benefits, albeit minor but still statistically significant, were seen following creatine supplementation in this young, healthy demographic, there remains a question as to whether creatine supplementation could impart an even greater benefit when applied in a clinical population suffering from CVD or a population already at risk of vascular dysfunction.

Age is a primary risk factor for CVD across the lifespan [117], partly due to the natural process of vascular aging characterized by reductions in NO [114], arterial stiffness [99], increase in ROS [83,118], ED [119], and inflammation [120]. Due to these age-related changes and the likelihood that creatine supplementation would be beneficial in older individuals, we conducted a small pilot study in 50–70-year-old individuals [121]. Four older adults were given creatine for five days at a loading dose of 4 x 5 g/day, and macrovascular endothelial function was determined via brachial artery flow mediated dilation (FMD). Although there was no statistical significance found, each individual showed a clinically relevant improvement in FMD% (+2.4 percentage units), which was similar to, or greater than, improvements in older individuals in response to drug treatment [122], other dietary supplementation [123], and exercise training [124]. Additionally, improvements in FMD% still remained once normalized to account for individualized shear stress. Despite the lack of control and small sample size, the indication of vascular improvement following just five days of creatine provides further indication of the potential for creatine to contribute to improved vascular health.

## 5. Potential Mechanisms of How Creatine May Improve Vascular Health

Despite the scarcity of literature exploring creatine’s physiological role within the vasculature, there are a number of ways in which creatine may be therapeutically beneficial for vascular health. Creatine may, for example, decrease oxidative stress and contribute to the antioxidant system, reduce circulating levels of homocysteine, and reduce chronic or acute inflammation.

### 5.1. Creatine, Oxidative Stress, and the Antioxidant System

Oxidative stress can be defined as a state of imbalance between the production of damaging free radicals and removal by natural antioxidant defenses [125]. Highly reactive, unstable free radicals can be formed from many compounds; however, those most common include reactive oxygen species (ROS) and reactive nitrogen species (RNS); or collectively, reactive oxygen and nitrogen species (RONS) [126]. Although in small amounts these radicals are necessary for various beneficial physiological actions [127], an abundance of RONS can cause irreversible damage to biomolecules, proteins, carbohydrates, lipids, ribonucleic acid (RNA), and deoxyribonucleic acid (DNA), underlying the development of many pathologies [83,128]. Fortunately, the human body possesses an antioxidant system to protect against free radical toxicity. The physiological antioxidant system is diverse with enzymatic and non-enzymatic processes, both serving to lower the oxidative potential of RONS through direct and in-direct mechanisms. Direct antioxidants, which are redox active, are sacrificed during the process in which they trap and deactivate RONS and must be replenished or regenerated [129]. Indirect antioxidants, on the other hand, may or may not be redox active and exert their antioxidant effects through the upregulation of cytoprotective proteins [129]. Despite the promise of this natural defense, the healthy function of the antioxidant system has been shown to diminish progressively as a result of aging [83,130], in addition to other factors such as poor nutrition [131] and lack of physical activity [132]. Considering the importance of antioxidants, the benefits of consuming antioxidant-containing supplements and their impact upon oxidative stress have been well researched [128,133,134]. Relatively new research indicates that creatine may also possess both indirect- and direct-antioxidant properties [10,11,135]. Considering that oxidative stress underlies many CVDs [134,136], if creatine could appropriately reduce oxidative stress, then creatine supplementation may be able to improve vascular health.

One of the first groups to investigate the antioxidant properties of creatine was Matthews et al. [137]. Using an animal model of Huntington’s disease, Matthews et al. examined whether creatine could protect against the formation of striatal lesions (tissue damage) in the brain, induced either by intrastriatal injections of malonate or intraperitoneal injections of nitropropionic acid (3-NP); both of which are inducers of neurotoxicity. It was reported that supplementation of 1% creatine for two weeks significantly reduced malonate-induced striatal lesions and reduced 3-NP-induced striatal lesion volume by 83%, indicating significant neuroprotection. It was also reported that supplemental creatine significantly increased striatal stores of PCr while preventing 3-NP-induced reduction in other energy metabolites. Finally, creatine significantly protected the animals against malonate-induced increases in the conversion of salicylate to 2,3-and 2,5-dihydroxybenzoic acid, which stands as a marker of hydroxyl free radical generation. Matthews et al. concluded here that creatine exhibited novel antioxidant properties and suggested that creatine supplementation could serve as a potential therapeutic strategy for neurodegenerative disorders characterized by oxidative stress.

Lawler et al. [10] was the first study to assess the hypothesis that creatine was capable of exerting direct antioxidant properties. Experiments were conducted using a highly controlled acellular setting to determine the antioxidant ability of varying doses of creatine on five ROS systems: xanthine oxidase for superoxide anions (O_2_^−^), H_2_O_2_, peroxynitrite (ONOO^−^), lipid peroxidation, removal of 2,2′-azino-bis3-ethylbenzothiazoline-6-sulphonic acid (ABTS^+^) cation radical, and tert-butyl-hydroperoxide (tBOOH). Antioxidant scavenging capacity (ASC) was also assessed. Lawler et al. reported that although creatine had no effect upon non-radical oxidants (H_2_O_2_ or tBOOH), creatine exhibited significant scavenging of ionized radicals such as ABTS^+^, O_2_^−^, and ONOO^−^. Additionally, there was a direct dose-response relationship found between creatine and total ASC. Despite this study being limited to a controlled acellular environment, these novel results clearly demonstrated the direct antioxidant capacity of creatine, thus prompting the need for further in vitro and in vivo investigation.

In an attempt to further identify the antioxidant potential of creatine in an in vitro setting, Sestili et al. [138] investigated the antioxidant potential of creatine on oxidatively injured animal (murine myoblasts–C2C12) and human (promonocytic–U937; umbilical vein endothelial cells–HUVEC) cultured cell lines. Cells were pretreated with varying doses of creatine before being treated by a variety of oxidative stressors (H_2_O_2_, tBOOH, or ONOO^−^) all capable of generating free radicals and inducing cell death. The authors reported that creatine treatment significantly attenuated the cytotoxic effects of each oxidative stressor, improving cell vitality in a dose-dependent manner. It was further found that creatine supplementation successfully increased intracellular contents of creatine but had no impact upon antioxidant enzymes. From these findings it was concluded that creatine was capable of exerting mild, yet significant, antioxidant activity. Interestingly, Sestili et al. also reported that antioxidant effects were abolished following the addition of a creatine uptake inhibitor, β-guanidinopropionic acid. This therefore suggests that the antioxidant effects of creatine are dependent upon intracellular creatine concentrations.

Mitochondria and mitochondrial DNA (mtDNA) are susceptible targets of free radical damage and mtDNA mutations are an underlying etiology of CVDs [139]. Guidi et al. [80] investigated the potential of creatine to serve as an antioxidant against oxidatively injured DNA. Cultured human umbilical vein endothelial cells (HUVECs) were pretreated for 24 h with varying doses of creatine before being treated with 200 μM of H_2_O_2_ to induce oxidative damage. The pretreated cells were then grown for a further 72 h while cell viability and DNA damage were assessed. Guidi et al. reported that cells pretreated with creatine had significantly increased viability following H_2_O_2_ insult, and that mtDNA was significantly protected from oxidative damage in comparison to controls. Guidi et al. concluded that creatine exhibited direct antioxidant effects, successfully protecting mtDNA against cytotoxicity induced by oxidative stress. These results, in addition to those previously reported, further support the notion that creatine could be a successful therapeutic strategy for combating oxidative stress-induced diseases.

The scientific evidence supporting creatine’s direct and indirect antioxidant properties does not stop with the above-outlined studies. Further evidence reported by Fimognari et al. [140], Rambo et al. [141], Sestili et al. [142], and Hosamani et al. [143] all support the contention that creatine protects cellular components against varying forms of oxidative stress. An in-depth review by Sestili et al. [11] further outlines creatine’s role as an antioxidant. Additionally, Meyer et al. [32] reported that activation of mtCKs by supplemental creatine contributed to sustained and efficient electron transport chain functioning, thereby reducing mitochondria-specific ROS production (H_2_O_2_) directly at the source. Although the majority of these studies were conducted in vitro or using animal models, there is evidence to suggest creatine offers analogous antioxidant protection in humans as well. For example, Rahimi et al. [18] reported a significant reduction in markers of lipid peroxidation and DNA oxidation in response to an acute anaerobic exercise stimulus following seven days of creatine supplementation (4 × 5 g/day) in young, resistance-trained males.

Although how mechanistically creatine exerts antioxidant effects is still speculative, there is evidence supporting that creatine can serve as a direct and indirect antioxidant. Supplements or foods rich in antioxidants have been shown to benefit a variety of at-risk populations such as the elderly [133,144], and have also been shown to have potential to help those suffering from CVDs that are characterized by vascular dysfunction and oxidative stress [145,146,147,148]. Further clinical trials are needed to assess the generalizability of the above findings and to explore the long-term benefits of creatine for combating oxidative stress-related CVD in humans.

### 5.2. Creatine and Homocysteine

Homocysteine is a sulfhydryl-containing amino acid and a byproduct of creatine synthesis [2]. Following the demethylation of S-adenosylmethionine (SAM) via GAMT, creatine and S-adenosylhomocysteine (SAH) are formed. SAH is then hydrolyzed into homocysteine (Hcy) via SAH hydrolase (SAHH). Elevated levels of Hcy have been associated with an increased risk for a variety of CVDs [149]. For example, a 5 μM increment in plasma Hcy has been associated with a 60% and 80% increase in the risk of coronary heart disease in both men and women, respectively [150]. Although increased plasma Hcy levels have been established as a potent independent risk factor for CVD development, the underlining mechanism in still largely unknown. It has been proposed, however, that the detrimental effects of increased plasma Hcy levels on vascular health are, in part, mediated by ROS accumulation and consequent reductions in NO bioavailability [151]. Considering that creatine synthesis consumes between 40–70% of the total labile methyl groups [152,153], it has been proposed that creatine supplementation may spare the investment of SAM, thereby reducing the formation of Hcy and lessening the risk of CVD development [154,155].

One of the first to test this hypothesis was Stead et al. who supplemented Sprague-Dawley rats with either creatine or GAA for two weeks [156]. Following supplementation, the authors reported that plasma Hcy was significantly increased (+50%) in rats supplemented with GAA, but significantly lower (−25%) in those supplemented with creatine. Steenge et al. similarly tested this hypothesis but did so using a cohort of healthy, young women [157]. Following supplementation (20 g/day for five days, 3 g/day for eight weeks), it was reported that those ingesting creatine alone exhibited a small, nonsignificant decrease in mean plasma Hcy concentrations (−0.3 ± 0.5 μmol/L); however, those ingesting creatine and partaking in RT showed a further reduction (−0.6 ± 0.5 μmol/L), yet this was still statistically insignificant. Although results from this early study lacked significance, it is possible that an increase in Hcy might be detected in less healthy individuals, such as those suffering from compromised or dysfunctional Hcy homeostasis.

Considering that hyperhomocysteinemia is prevalent in more than 85% of patients with end-stage renal disease, Taes et al. investigated whether creatine supplementation could reduce plasma concentrations of Hcy in an animal model of uremia [158]. Authors reported that nephrectomized animals displayed the highest level of plasma Hcy; however, those fed supplemental creatine showed a significantly lower level of plasma Hcy in comparison to those fed a control diet (12.1 ± 2.4 μmol/L vs. 15.4 ± 1.7 μmol/L). More than a decade later, Deminice et al. used Walker-256 tumor-bearing rats, comparably presenting with hyperhomocysteinemia, to determine whether creatine supplementation could attenuate plasma levels of Hcy [159]. Analogous to that found by Taes et al., Deminice reported that creatine supplementation resulted in a significant reduction in plasma Hcy in comparison to those receiving a control diet (6.3 ± 0.9 μmol/L vs. 10.3 ± 1.5 μmol/L). These findings further supported those previously reported by Deminice, in which decreases in Hcy and markers of oxidative stress where seen following four weeks of creatine supplementation in healthy rats [160].

Although creatine supplementation displays Hcy-lowering potential in varying animal models, studies using human subjects still remain relatively inconclusive. Korzun et al. in a human trial of young, healthy subjects found that creatine supplementation taken in conjunction with a multi-vitamin for four weeks resulted in a statistically significant reduction in total plasma Hcy, in comparison to a control group consuming the multi-vitamin only (−0.9 μmol/L vs. + 0.2 μmol/L) [13]. The authors suggested from these results that creatine supplementation may be effective for lowering Hcy, but only when introduced as an adjuvant supplement. Similarly, Bereket-Yücel et al. reported that creatine supplementation (25 g/day for first five days, 5 g/day thereafter for eight weeks) in addition to RT lowered plasma Hcy levels in young males (pre = 12.66 ± 5.89 μmol/L vs. post = 9.33 ± 4.60 μmol/L) [14]. No reductions in Hcy were reported for either the placebo or placebo + RT groups; however, the lack of a creatine-only group limits the ability to conclude whether creatine lowered plasma Hcy independently, or whether the addition of RT augmented this effect. In addition to these studies, Van Bavel et al. [12] found that creatine supplementation (5 g/day for three weeks) significantly reduced plasma Hcy levels in strict vegans with hyperhomocysteinemia, in comparison to a placebo.

In contrast, a comparable number of studies report either little or no reduction in Hcy following creatine supplementation. Peters et al. reported that creatine supplementation (3 g/day for 12 weeks) in Bangladeshi adults did result in a significant reduction in GAA, representative of a decrease in the first step of creatine synthesis; however, no reduction was reported for Hcy, SAM, or SAH [161]. A study by Deminice et al. utilizing young soccer players further reported that seven days of creatine supplementation (0.3 g/kg body weight) did not prevent the increase in plasma Hcy induced by an acute bout of exercise [162]. Furthermore, Taes et al., who had previously reported Hcy lowering effects of creatine supplementation in an animal model of uremia, reported no reduction in Hcy following supplementation (2 g/day for four weeks) in patients suffering from chronic hemodialysis [163]. Even more controversially, Jahangir et al. [164] and Shelmadine et al. [165] both reported significant increases in Hcy levels following creatine supplementation.

Although in vivo studies have provided evidence to suggest the Hcy-lowering properties of creatine [156,158,159,160], clinical trials are relatively inconsistent with some supporting [12,13,14,157] and some opposing [161,162,163,164] this hypothesis. This therefore makes it difficult to confidently conclude that creatine supplementation lowers CVD risk by reducing Hcy in humans. The variations between studies could be due to differing disease state, age, dosing strategy, time frame, or inclusion of additional stimuli such as RT or multivitamins. Despite the inconsistencies found between clinical trials, there is still evidence supporting the role of creatine in Hcy metabolism. Considering that CVD develops over time and the apparent lack of longitudinal studies, there is a clear need for future investigations focusing on the long-term impact of creatine supplementation on Hcy. Furthermore, few studies have investigated the impact of creatine on Hcy within older adults specifically, and considering the natural increase in plasma Hcy seen progressively with age [166,167,168], one might hypothesize a greater benefit of creatine within this population as compared to younger individuals.

### 5.3. Creatine and Inflammation

Inflammation, or the presence of chronic inflammatory markers, has been long associated with CVD [20,169]. Inflammation can detrimentally impact vascular function, contributing to the manifestation and progression of ED, and creating an environment that fosters the development of vascular pathologies. Although the literature is limited regarding the impact of creatine on inflammation, there are a few reports that suggest potential anti-inflammatory effects of creatine.

Madan and Khana were amongst the first to investigate the potential anti-inflammatory role of creatine. To determine the ability of creatine to function as an anti-inflammatory agent, Madan and Khana utilized a rat model of carrageenan-induced acute inflammation: one of the most common models utilized in inflammatory research [170]. The injection of carrageenan into the paw causes an acute and local inflammatory response characterized by edema and the release of histamine, serotonin, bradykinin, prostaglandins, and cytokines such as interleukin-1beta (IL-1β), IL-6, IL-10, and tumor necrosis factor alpha (TNF-α). Using this model, Madan and Khana reported that those animals treated with an intraperitoneal injection of creatine showed significantly reduced paw swelling and edema [171]. A follow-up study was later conducted to determine the efficacy of creatine for the mediation of acute and chronic inflammation, as well as the potential for creatine to serve as a local analgesic. Utilizing the same animal model, it was similarly reported that treatment with creatine successfully attenuated inflammatory symptoms to the same extent as a control non-steroidal anti-inflammatory drug (NSAID-phenylbutazone) [172]. The authors further examined the application of creatine for defending against other edema models including formaldehyde-induced arthritis, and analogous to previous findings, creatine proved again to be a beneficial anti-inflammatory agent [171,172,173]. Despite these findings being limited to localized inflammation in animals, the above studies served as the impetus for further examination into the anti-inflammatory potential of creatine.

Nomura et al. [17] expanded upon these early findings and investigated the impact of creatine supplementation on EC stores of creatine, PCr and ATP, in addition to the anti-inflammatory activity of creatine. Using pulmonary ECs in culture, the addition of 0.5 mM of creatine to the culture medium was found to significantly increase both intracellular creatine and PCr stores in ECs, with cellular uptake being mediated by an evidential CRT. The addition of 5 mM of creatine also significantly suppressed the endothelial permeability induced by serotonin and H_2_O_2_, indicating improved membrane stability and reduced EC leakiness. Furthermore, when assessing varying cell adhesion molecules associated with inflammation, supplemental creatine of 5 mM significantly reduced neutrophil adhesion to ECs, while supplementation of just 0.5 mM of creatine inhibited the expression of Intercellular Adhesion Molecule-1 (ICAM-1) and E-selectin on ECs. Considering that elevated levels of ICAM-1 [174] and E-selectin [175] and increased endothelial permeability or “leakiness” [176,177] have all been linked to ED and CVDs, these results suggest promising potential for creatine to serve as an anti-inflammatory aid and to provide vascular protection.

Creatine has also been shown in many studies to help attenuate the inflammatory response to immune stressors, such as exercise-induced muscle damage. Santos et al. [178] evaluated the impact of creatine supplementation (20 g/day) taken for five days prior to a 30 km race event on inflammatory markers. The authors reported that creatine supplementation attenuated prostaglandin E_2_ (PGE_2_) and TNF-α concentrations by 60.9% and 33.7%, respectively, in comparison to the placebo group that presented with significant increases in all markers. Similarly, Bassit et al. [16] used an analogous creatine supplementation protocol five days prior to a half Iron Man event. The authors reported that those individuals who ingested creatine showed a markedly reduced increase in plasma levels of TNF-α, PGE_2_, interferon-α (INF-α), and IL-1β, in comparison to placebo at both 24- and 48-h post-event. Further supporting these findings, Deminice et al. [179] showed that seven days of creatine supplementation (0.3 g/kg body weight) in young soccer players abolished the increase in TNF-α seen following a repeated sprint exercise, in comparison to placebo. Although in contrast Silva et al. [180] reported no reduction in inflammatory markers following eccentric contraction in rats, the majority of the above evidence suggests that creatine attenuates the pro-inflammatory response to exercise, therefore exhibiting potential anti-inflammatory properties.

Despite the lack of studies on the anti-inflammatory potential of creatine as it specifically pertains to the human vasculature, there is evidence to suggest that creatine can impact the inflammatory response [171,172,173], and reduce circulating levels of damaging cytokines [17] in response to various stressors such as exercise [181]. Considering the association between global inflammation and CVD [20,169], there is a clear need for further research focusing on the application of creatine for the management of inflammation. Furthermore, perhaps more specifically, there is a need for studies to utilize populations that present with underlining chronic inflammation, such as the elderly [120] or those with atherosclerosis [182], in order to truly determine the anti-inflammatory ability of creatine and the therapeutic value it may have for vascular health.

## 6. Conclusions

Although creatine was extracted in 1832, creatine supplementation did not become mainstream or publicly well-known until the 1990s when two Olympic gold medalists credited the addition of creatine supplementation as part of their sporting success [183,184]. Since then there has been an extensive and ever-growing body of literature surrounding the efficacy and application of dietary creatine supplementation. It is clear that the addition of supplementary creatine to the diet can exert beneficial ergogenic effects, enhancing a variety of sport performance variables such as lean mass, fatigue resistance, power output, and intermittent high-intensity exercise capacity. Furthermore, it is evident that creatine plays a quintessential role in functional bioenergetics, metabolism, and overall cellular health. From a clinical perspective, these findings suggest an exciting potential for creatine supplementation as an adjuvant therapy for a multitude of pathologies. Evidence of therapeutic benefits induced by creatine have been reported in pathologies such as myopathies, chronic kidney disease, neurodegenerative disorders, joint syndromes, and type 2 diabetes. Additionally, creatine has been shown to function as an antioxidant, protecting against damage caused by free radicals. Unsurprisingly, the clear widespread potential of this simple supplement has led many to propose that this natural compound is arguably one of the most promising, pleiotropic, nutritional supplements in the therapeutic field. In addition, over the course of decades of research, creatine has been found to exhibit an excellent safety profile, with minimal side effects presenting after acute or chronic supplementation, in moderate or large doses, in a variety of populations from young to old [5,185].

Despite the strong evidence for the clinical application of creatine, much of this evidence has been built upon in vitro and animal models with a limited number of clinical trials attempting to expand upon this evidence. Additionally, the mechanisms by which creatine exerts beneficial effects are still relatively unknown; therefore, this warrants further investigation to provide necessary insight into potential mechanisms of action. Furthermore, there is still much yet to be discovered in regard to whether creatine could provide therapeutic benefit within other specific clinical diseases, perhaps as an adjuvant therapy for those pathologies characterized by bioenergetic disturbances, inflammation, or oxidative stress, such as CVDs.

**Figure 2:**
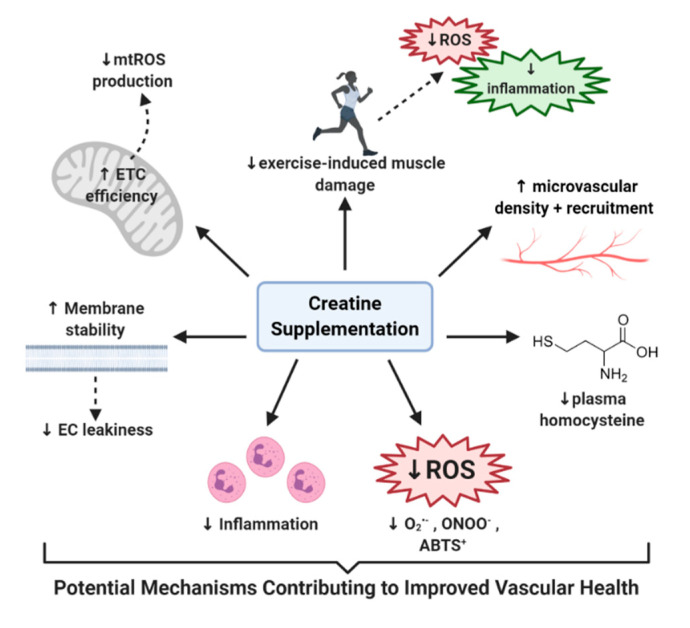
Potential mechanisms contributing to improved vascular health: ROS = reactive oxygen species, ABTS+ = 2,2’-azino-bis3-ethylbenzothiazoline-6-sulphonic acid, ETC = electron transport chain, mtROS = mitochondrial specific ROS, EC = endothelial cell, O_2_^−^ = superoxide, ONOO^−^ = peroxynitrite.

In summary, throughout this review we have highlighted studies that have not only shown potential benefit of using creatine to improve vasculature function, but have also elucidated the potential of creatine to alleviate the various factors that contribute to the development of CVD (Figure 2). For example, creatine has shown potential to: (1) help decrease Hcy levels in those with dysfunctional Hcy homeostasis [12,154], (2) increase EC-specific creatine stores [17,27], (3) quench damaging free radicals such as superoxide and peroxynitrite, which have long been associated with CVD [10,18,138], (4) manage the inflammatory response [17,173,181], (5) improve EC membrane stability and decrease EC leakiness [17,150], (6) preserve the integrity and efficiency of mitochondria and reduce mtROS production [32], (7) reduce hemodynamic and inflammatory responses to exercise [16,108,178], and (8) increase microvasculature density, recruitment, and vasotone [12,112]. Therefore, it is clear that further disorder-specific randomized control trials must be conducted, using populations who are at risk of vascular deterioration and complications, to determine the full therapeutic potential of creatine supplementation in the fight for vascular health.
